# Patterns of gene expression in microarrays and expressed sequence tags from normal and cataractous lenses

**DOI:** 10.1186/1479-7364-6-14

**Published:** 2012-09-01

**Authors:** Konstantinos Sousounis, Panagiotis A Tsonis

**Affiliations:** 1 Department of Biology and Center for Tissue Regeneration and Engineering, University of Dayton, Dayton, OH, 45469-2320, USA

**Keywords:** Lens, Microarray, Cataract, EST

## Abstract

In this contribution, we have examined the patterns of gene expression in normal and cataractous lenses as presented in five different papers using microarrays and expressed sequence tags. The purpose was to evaluate unique and common patterns of gene expression during development, aging and cataracts.

## Introduction

The lens is the organ inside the eye dividing it into two chambers: the aqueous humor in the anterior side and the vitreous humor in the posterior side. Lens focuses light from the environment to the retina for correct vision. The cornea is the most anterior surface that is in contact with the aqueous humor and the environment. Both the cornea and lens originated from the surface ectoderm during development. During gastrulation, the ectoderm is patterned to be the presumptive lens ectoderm and retina. Neurulation makes the presumptive retina tissues invaginate and become part of the neural ectoderm, which will form the optic vesicle, and it interacts with the presumptive lens ectoderm in later stages of neurula. In this stage, the fate of the presumptive lens ectoderm is already determined, and the induction of lens begins with the lens placode, which will evaginate to form the lens vesicle. The lens vesicle will give rise to mature lens, which is polarized with lens epithelium in the anterior side and differentiated lens fibers in the posterior (for reviews see [1-5]).

During all these steps, signaling pathways, transcription factors and gradients of growth factors play essential role in patterning the different tissues, making them competent in forming the appropriate tissues, stabilizing their fate during the development of the organism and finally differentiate them to play their designated role [6,7]. Some of these factors are paired box protein 6 (Pax6) [8-35], sex determining region Y-box 1 (Sox1), Sox2, Sox3 [16,28,31,36-43], sine oculis homeobox homolog 3 (Six3) [17,32,44-46], pituitary homeobox 3 (Pitx3), [47-54], prospero homeobox protein 1 (Prox1), [23,55-60], transcriptional factor of the fork head family (Fox3e), [61-64], leucine zipper transcription factors of the maf family [65-70], fibroblast growth factors (FGF) [71-99], fibroblast growth factor receptors (FGFR) [91,94,100-105], bone morphogenetic proteins 4 (Bmp4), bone morphogenetic protein 7 (Bmp7) [106-109] Bmps/TGFb and their receptors [110-117], extracellular matrix (fibrin, laminin and fibronectin) [118-121], integrin signaling [122-124], insulin [125,126], insulin-like growth factor-1 (IGF-1) [127] and activator protein 2 (AP2) [128,129].

Functional analysis for most of these factors using transgenic animal models revealed that they associate with known diseases. Disruption of one or more of these factors from their normal role during development leads to abnormal phenotypes. In lens, most of the abnormal phenotypes result to cataract, a term which is used for all the situations where light cannot pass through the lens or it is scattered but not focused on the retina. Crystallins, the most abundant proteins in the lens, which also make the lens transparent, are disorganized during cataract. Cataract has many forms and causes, and it can change depending on the age and the region of the globe [130-135]. Classification depends on the patient's age, location in the lens, maturity stage and cause. Cataract can be formed together with other developmental disorders depending on mutations of factors that play roles in the differentiation of the lens, such as Pitx3 [136], crystallins, ferritin, connexins, aquaporins, LIM2, filensin, phakinin, heat shock transcription factor 4, Foxe3, Chx10, Maf family, Pax6, Gcnt2, Chmp4b (reviewed [137]), extracellular matrix (Capsule), fibrillin-1 [138], lysyl oxidase-like 1 (Loxl1) [139-141], laminin subunit b2 (lam-b2) [142], collagen IV a1 (col4a1) [143], collagen IV a3 (col4a3), collagen IV a4 (col4a4), collagen IV a5 (col4a5) [144], collagen XVIII [145], fibronectin [146], Sparc [147,148], collagen type I [149-151], FGF receptors [105], matrix metalloproteinases 2 and 9 [152,153] and integrins [154]. Similarly, metabolic diseases like diabetes, which leads to accumulation of glucose products inside the lens, can result in cataracts [155,156]. Also, there are certain genetic syndromes that contribute to cataract formation, such as Nance-Horan syndrome [157,158], Lowe syndrome (neurofibromatosis type 2) [136], hyerferritinemia cataract syndrome, Marfan syndrome [138], pseudoexfoliation syndrome [159], Pierson syndrome, Alport's syndrome [144,160], Knobloch syndrome [145] and phacotoxic uveitis [161,162]. However, despite the high percentage of success in cataract surgeries, complication can result in secondary cataract. The most important mechanism that is recognized to play a role in secondary cataract formation is the Epithelial-Mesenchymal Transition (EMT). This pathway is heavily studied because of its role in different kinds of situations, pathological or developmental [151,163,164]. TGFb is the soluble protein that activates this pathway in the lens epithelial cells to differentiate to fibro-myoblasts and not to lens fiber cells, which eventually leads again to opacification of the lens.

Age is a factor that influences many molecular, genetic and metabolic networks. For example, in lens, the lens capsule becomes thicker with age [165-167]. The lens loses functional properties and changes its protein contents. Membranes become harder, and that can influence the function of transmembrane proteins playing a role in cell-cell communication or homeostasis like aquaporins or connexins. The appearance of an internal lens barrier may play a role in preventing the smooth transportation of molecules in and out of this avascular tissue [168,169]. This might result in accumulation or lack of certain molecules, which can result in cataract. Furthermore, modifications in proteins appear in aged lens. These modifications can be cleavage of structurally important proteins like crystallins or modifications in certain residues, which can lead to altered interactions with other molecules. Finally, the lens' UV defenses are lost with age. UV can cause many structural alterations to proteins, resulting in lens opacification [170,171]. Oxidative stress is very important in cataract formation and is extensively studied [172]. Reduced levels of glutathione are the major cause of age-related nuclear cataract [130,171,173,174]. Additionally, proteins are oxidized, modified and cross-linked, so they lose their functional properties. Also, hyperoxide with metals can influence molecular and homeostatic regulation in the lens, resulting in age-related nuclear cataract (see review [171]).

As discussed above, there are mechanisms that induce lens from the ectoderm. Signaling pathways, molecular interactions and cell communication create the normal lens in the eye cup. It has the mechanisms to repair genetic and molecular damage from light. Also, this avascular tissue needs mechanisms to keep homeostasis. All these networks must restart if the tissue is injured or if it regenerates a part of it. During regeneration, all the cascades must be enabled again. In the past years, methods, such as microarrays, to analyze genes and gene expressions in a high-throughput approach have been utilized. Genes expressed in developing lens, lens during regeneration, aging lens and lens with cataract have been analyzed by these approaches. The purpose of this contribution is to summarize and compare the genomic analysis that has been performed in these different conditions. Most of the times, the data generated from this kind of analysis make it difficult to interpret and even more when different data sets are compared. Here, we have compared data sets from five different studies, and we have identified similar and different patterns between the different tested groups that were generated from genomic analysis.

## Analysis

We have selected five papers dealing with microarray data and expressed sequence tag analysis. There are, of course, other papers in the literature that use high-throughput approaches to study gene expression in the lens [34]. However, we have decided to concentrate to these papers because of the conditions of the lens used (age-related cataract, Sparc-null mice and regenerating lens).

### Study #1: ‘Expressed sequence tag analysis of adult human lens for NEIBank Project: over 2000 non-redundant transcripts, novel genes and splice variants’

Two adult (40 years old) human lenses were used, and two libraries were created: (by) non normalized and (fs) normalized. Genes that are expressed in the lens or are abundant in the lens were found [175]. However, there was no mention of any comparison with other tissues. The data we used have been taken from the following tables of the paper:

· Table one: The twenty five most abundant transcripts in the unamplified lens library (by)

· Table two: Cytoskeleton related transcripts from the combined lens libraries (by + fs)

· Table three: Oxidation related transcripts observed in by and fsEST collections

· Table four: Protease related transcripts

· Table five: Transcription factors

· Table six: Transcripts for growth factors, cytokines and growth factor related genes in lens libraries.

· Table seven: Apoptosis related transcripts in lens

### Study #2: ‘Expression profiling and gene discovery in the mouse lens’

Mouse lenses of different ages and non-lens tissues to compare differentially regulated genes were used [176]. The data we used have been taken from the following tables of the paper:

· Table two: ONTO-express analysis of the 1,668 genes expressed at above background plus 2 SD levels in at least one of the lens

· Table three: A list of the 50 most highly expressed genes identified in the lens samples

· Table four: Genes potentially preferentially expressed in lens compared to non lens samples as determined by *K*-means clustering

· Table six: Apoptosis genes expressed in the lens

· Table seven: Additional novel and uncharacterized genes expressed in the lens confirmed by Reverse transcription-Polymerase chain reaction (RT-PCR)

· Table eight: Additional known genes expressed in the lens confirmed by RT-PCR

### Study #3: ‘Gene expression changes during cataract progression in Sparc null mice: differential regulation of mouse globins in the lens’

Microarray analysis of adult lenses from Sparc knockout mice on two strain backgrounds was used [177]. The data we used have been taken from the following tables of the paper:

· Table four: Confirmed genes: Nine month Sparc^tm1cam^ 129 Sv/Ev lenses versus 129 Sv/Ev controls

· Table five: Confirmed genes: Nine month Sparc^tm1cam^ 129/Ev/Mf1^GPI-BB^ lenses versus Mf1^GPI-BB^ controls

### Study #4: ‘Identification and functional clustering of global gene expression differences between human age-related cataract and clear lenses’

Microarray analysis was used to find differences between age-related cataract and clear lenses in human [178]. The data we used have been taken from the following tables of the paper:

· Table one: Genes exhibiting differential expression in cataract relative to clear lenses

· Table continued: Genes exhibiting decreased expression in cataract relative to clear lenses

### Study #5: ‘Gene expression and discovery during lens regeneration in mouse: regulation of epithelial to mesenchymal transition and lens differentiation’

Microarray analysis of mouse lens was used during regeneration after surgery [179]. The data we used have been taken from the following tables of the paper:

· Table one: Top 50 genes with the greatest increase in relative mRNA expression levels of regenerating lens compared to intact control lens 1, 2 and 3 weeks post-extracapsular surgery. For a better visual presentation of the regulated genes at 1, 2 and 3 weeks, the times that each gene is highest is marked in red.

· Table two: Top 50 genes with the greatest decrease in relative mRNA expression levels of regenerating lens compared to intact control lens 1, 2 and 3 weeks post-extracapsular surgery. For a better visual presentation of the regulated genes at 1, 2 and 3 weeks, the times that each gene is highest is marked in red.

· Table four: The gene cluster displaying a weak uniform increase in relative mRNA expression levels of regenerating lens compared to intact control lens 1, 2 and 3 weeks post-extracapsular surgery. For a better visual presentation of the regulated genes at 1, 2 and 3 weeks, the times that each gene is highest is marked in red.

· Table six: The gene cluster displaying a strong uniform increase in relative mRNA expression levels of regenerating lens compared to intact control lens 1, 2 and 3 weeks post-extracapsular surgery. For a better visual presentation of the regulated genes at 1, 2 and 3 weeks, the times that each gene is highest is marked in red.

· Table eight: The gene cluster displaying a strong increase in relative mRNA expression levels of regenerating lens compared to intact control lens 1, 2 and 3 weeks post-extracapsular surgery. For a better visual presentation of the regulated genes at 1, 2 and 3 weeks, the times that each gene is highest is marked in red.

· Table ten: The gene cluster displaying a weak decrease in relative mRNA expression levels of regenerating lens compared to intact control lens 1, 2 and 3 weeks post-extracapsular surgery. For a better visual presentation of the regulated genes at 1, 2 and 3 weeks, the times that each gene is highest is marked in red.

· Table twelve: The gene cluster displaying a strong uniform decrease in relative mRNA expression levels of regenerating lens compared to intact control lens 1, 2 and 3 weeks post-extracapsular surgery. For a better visual presentation of the regulated genes at 1, 2 and 3 weeks, the times that each gene is highest is marked in red.

## Presentation of analysis

The data taken from the tables above were sorted and categorized depending on their expression in the different conditions that we examine. In our tables, the columns represent the following:

· *Column 1: Name.* Name of genes are the same with those taken from the tables of the different papers. If the same names were identified, they were fused in one row.

· *Column 2: Accession number*. Accession numbers are the ones from the selected papers. They can be from gene, protein or expressed sequence tags (EST) databases. If two or more genes (same gene, with a different name) are presented as one in a row, more accession numbers may appear in the corresponding column. In parenthesis are the new accession numbers that are assigned to the different genes, without omitting the old accession number from the original paper.

· *Column 3: Lens*. Genes found in the lens in general. Red, information from the paper ‘Expressed sequence tag analysis of adult human lens for NEIBank Project: over 2000 non – redundant transcripts, novel genes and splice variants’ [175]. Dark red, information from the paper ‘Expression profiling and gene discovery in the mouse lens’ [176] for genes that are not shown to be differentially expressed between lens and non-lens tissues.

· *Column 4: More in lens*. Genes that are found to be differentially expressed between lens and non-lens tissues. Red, information from the paper ‘Expression profiling and gene discovery in the mouse lens’ [176] for genes that are differentially expressed between lens and non-lens tissues. Yellow, information from the same paper but for genes that are not differentially expressed between lens and non-lens tissues.

· *Column 5: Up in Sparc*. Genes that are up-regulated in Sparc-null mice compared to normal controls. Red, information from the paper ‘Gene expression changes during cataract progression in Sparc-null mice: differential regulation of mouse globins in the lens’ [177].

· *Column 6: Down in Sparc*. Genes that are down-regulated in Sparc-null mice compared to normal controls. Red, information from the paper ‘Gene expression changes during cataract progression in Sparc-null mice: differential regulation of mouse globins in the lens’ [177].

· *Column 7: Up in cataract*. Genes that are up-regulated during age-related cataract compare to clear lenses. Red, information from the paper ‘Identification and functional clustering of global gene expression differences between human age-related cataract and clear lenses’ [178].

· *Column 8: Down in cataract*. Genes that are down-regulated during age-related cataract compared to clear lenses. Red, information from the paper ‘Identification and functional clustering of global gene expression differences between human age-related cataract and clear lenses’ [178].

· *Column 9: Up in regeneration*. Genes that are up-regulated during lens regeneration compared to intact lenses. Red, information from the paper ‘Gene expression and discovery during lens regeneration in mouse: regulation of epithelial to mesenchymal transition and lens differentiation’ [179].

· *Column 10: Down in regeneration*. Genes that are down-regulated during lens regeneration compared to intact lenses. Red, information from the paper ‘Gene expression and discovery during lens regeneration in mouse: regulation of epithelial to mesenchymal transition and lens differentiation’ [179].

· *Column 11 to 16* (Only present in Additional file 1: Table S2). Categories for gene functions. Red, if there was information from the five papers used.

## Discussion

From the analysis we have performed, we have divided the regulated genes into eight major groups for our discussion.

### Crystallins-heat shock proteins

Crystallins and heat shock proteins (Additional file 2: Table S4) comprise the main structural part of the lens. They have protective antioxidant properties and work as chaperones. Crystallins account for approximately 90 % of total lens proteins [180]. They make the lens transparent in order for light to pass through and focus on the retina. Most of the different kinds of crystallins are present in the lens, as shown in Additional file 2: Table S4, column 3. Some of them are found up-regulated in the lens than in other tissues (crystallin beta A1, beta A2, gamma A, gamma C, gamma E; Additional file 2: Table S4, column 4). During cataract, crystallins are disrupted; cross-linking between them results in aggregation, insolubility and opacification of the lens. Most of the crystallins are down-regulated during age-related cataract, as shown in Additional file 2: Table S4, column 6. Among them, crystallin alpha A is a protein associated with known cases of cataract formation due to a mutation that results in an early stop codon in the gene [181] or other mutations that affect its interactions [182-189]. Furthermore, crystallin alpha B does not show any change during age-related cataract (Additional file 2: Table S4, columns 8 and 7), which is consistent with the fact that it has been shown to cause myopathy through other effects [190,191]. However, cataract has been associated with mutated crystallin alpha B in humans [192-196]. Beta crystallins are down-regulated during cataract (Additional file 2: Table S4, column 8), and they have a known role in cataract formation [197-215]. Crystallins gamma A and gamma D are also found in cataracts [216-234] and are down-regulated during age-related cataract (Additional file 2: Table S4, column 8). Some of the crystallins are down-regulated during regeneration of the lens from the lens capsule that is left behind, probably depending on the emergence of EMT in the early stages. Thus, crystallins that are found in mature lens, especially gamma crystallins, are down-regulated during regeneration (Additional file 2: Table S4, column 10). However, early crystallins are not up-regulated except a cytosolic thyroid hormone-binding protein, crystallin mu (Additional file 2: Table S4, column 9).

Heat shock proteins have structural and sequence similarities with crystallins and play transcriptional, structural and, most importantly, protective roles in the lens. Heat shock proteins are chaperones, and they protect the lens from oxidation and stress [235,236]. Some of them are found in the lens, as shown in Additional file 2: Table S4, column 3, but they do not seem to be significantly differentially expressed between the lens and other tissues (Additional file 2: Table S4, column 4). Similarly with crystallins, they are down-regulated during age-related cataract and regeneration (Additional file 2: Table S4, columns 8 and 10).

Overall, the comparison of the datasets clearly shows that crystallins malfunction during the different types of cataract except Sparc-related cataract. During age-related cataract and regeneration, crystallins are mostly down-regulated. There is an apparent connection between cataract and crystallins from all the studies that are performed in this field. The data are consistent, and they link the abnormal regulation of crystallins as a key player for cataract formation (Additional file 3: Table S5).

### Cytochrome

Cytochrome (Additional file 4: Table S6) is a protein related to electron transfer chain in the mitochondria, and it has been linked to stress [237]. Light induces stress response in the lens due to oxidation. This can be the reason why there are a lot of cytochrome oxidases present in the lens (Additional file 4: Table S6, column 3). The regulation of cytochrome oxidases is disrupted due to stress in the eye after surgically removing the lens fibers, a procedure that leads to regeneration-EMT response in the lens (Additional file 4: Table S6, columns 9 and 10).

Other than mitochondrial stress-related cytochrome oxidases, cytochromes of the P450 family seem to play a role in cataract formation. These proteins that are located in the endoplasmic reticulum can oxidize different kinds of substrates. Their regulation is disrupted similarly like cytochrome oxidases (Additional file 4: Table S6, columns 9 and 10). Studies show that cytochrome P450 is directly linked to cataract formation after metabolizing acetaminophen [238]. In addition, it is blamed for initiating cataract formation [239].

### Transcriptional factors

Many transcriptional factors (Additional file 5: Table S7) are present in the lens, as shown in Additional file 5: Table S7, column 3. Many of them can be found in all tissues in order to maintain housekeeping functions (ATF4, apoptosis antagonizing transcription factor, bHLH transcriptional factors, general transcriptional factor II and III, and transcriptional elongation factors). Others, as discussed in the introduction, are transcriptional factors during development that define the different regions of the optic cup which, with appropriate interactions, create a functional eye. The expression of these transcriptional factors also persists after the eye is formed. The lens specific transcriptional factors define the lens epithelium, the transition zone and the lens fibers [1,2,4]. These, important for the integrity of the lens transcriptional factors, are not found in age- or Sparc-related cataract or regeneration (Additional file 5: Table S7, columns 5, 6, 7, 8, 9 and 10). Mutated transcriptional factors can result in cataract formation along with other effects, but these situations are not part of the cataract cases we examine, which are caused by age, the lack of a glycoprotein (Sparc) or the surgically removed lens fibers.

As mentioned earlier, studies implicate transcriptional factors with cataract formation. These studies include human patients. To sum up, transcriptional factors are difficult candidates in studying cataract formation during aging or EMT because they are involved in many different aspects of cell physiology.

### Immunity

The lens is an avascular organ. Consequently, it is not in direct contact with blood flow. Thus, it is not surprising to see that proteins playing a role in the immune system are not expressed in the lens (Additional file 6: Table S8, column 3). However, two of them differentially show expression between the lens and other tissues (CD24 antigen and IL2-inducible T cell kinase; Additional file 6: Table S8, column 4). After surgically removing the lens fiber, which represents cataract formation through activation of EMT, many proteins that activate inflammation response, like complement components, are present, as seen in Additional file 6: Table S8, column 9. Interestingly a C5 antagonist has been found to be beneficial by inhibiting EMT in a lens regeneration model, even though the receptors involved are not known [240]. This implicates an inflammatory immune response [240]. On the other hand, during age-related cataract, there are a few proteins that are actually down-regulated (Additional file 6: Table S8, column 8), which might mean that the regulation of the immune system may play a role in maintaining the opacity of the lens. Sparc-null-related cataract shows up-regulation of complement components (Additional file 6: Table S8, column 5) that is linked to cell lysis, which is observed during Sparc-null cataract at the later stages.

Surgical removal of the lens fibers is a process that will trigger the immune system. The blood near the area of the damage brings molecules playing role in immune response. Also, white blood cells secrete other cytokines. These signals might promote the EMT process and play a role during age-related cataract. Oxidative stress is increasing with age, which in turn can trigger an inflammation response in neighboring tissues.

### Growth factors

Growth factors (Additional file 7: Table S9) are small proteins that bind to certain receptors and activate specific downstream cascade events. Gradients of growth factors are regulating the correct gene expression and patterning in the lens [1,3]. All the major growth factors are present in the lens (Additional file 7: Table S9, column 3), and there is not much variation in expression during age- or Sparc-related cataract or regeneration (Additional file 7: Table S9, column 5, 6, 7, 8, 9 and 10). These molecules, if not produced in the lens, must diffuse from the other parts of the eye like the cornea and retina and pass through the extracellular matrix of the lens capsule in order to reach the lens epithelium or fibers. During aging, the lens capsule is changing, so the regulation in the capsular bag level is reduced. This disrupts the accommodation of the lens and can lead to presbyopia with age or interfere with metabolic processes in the lens [153,171,241,242]. FGFR proteins are very important for the lens to sense the FGF gradient in the optic cup and have known roles during cataract formation without showing any change during age-related cataract (Additional file 7: Table S9, columns 7 and 8). TGFb, a molecule that plays the major role in EMT, and proteins that interact with it, such as latent TGFb-binding proteins, are up-regulated during regeneration (Additional file 7: Table S9, column 9). There might not be much about Sparc-null-related cataract and growth factor regulation (Additional file 7: Table S9, columns 5 and 6), but Sparc is a very important protein of the extracellular matrix. It can regulate various growth factors including FGF2, VEGF and PDGF [243]. This indicates that the cataractous lens might not differentially express growth factors, but it might respond differently to growth factors that reach the lens from other parts of the eye.

Overall, growth factors play an important role in cataract formation. They are directly implicated to secondary cataract formation through EMT where TGFb is the key player. For age-related cataract and Sparc-null cataract, growth factors seem to play a secondary role.

### Metalloproteinases/cathepsins

In the lens, there is a lot of extracellular matrix, which has to be maintained in the appropriate proportion of proteins in order to serve its role as the first barrier for molecule trafficking. Extracellular matrix is replaced over time [244]. Metalloproteinases are proteins that can degrade extracellular matrix composed of collagen, fibrin, various receptors, etc. As shown in Additional file 8: Table S10, column 3, there are many kinds of metalloproteinases present in the lens. During regeneration, the lens epithelial cells proliferate and migrate in order to create again the fibers. In order for the remaining lens epithelial cells to accomplish that, they have to degrade the extracellular matrix that has covered all the area in the eye after the surgical operation. They also have known roles in regeneration in various systems [245-251]. Additional file 8: Table S10, column 9 shows metalloproteinases and cathepsins that are up-regulated during regeneration. On the other hand, tissue inhibitors of metalloproteinases are up-regulated too (Additional file 8: Table S10, column 9). The correct regulation of extracellular matrix degradation is essential for the regeneration of the lens. A not so well-known role of metalloproteinases is the relationship they have with cataract. There are reports showing that the disruption of normal regulation of metalloproteinases can lead to cataract [252-254]. As shown in Additional file 8: Table S10, column 8, some metalloproteinases are down-regulated during age-related cataract. Interestingly, expression of matrix metalloproteinases is not altered in Sparc-null cataract even though the composition of the extracellular matrix in these lenses is altered.

Matrix metalloproteinases and cathepsins can be an area of investigation since they are found to be responsible for cataract formation. Inhibition of migration of epithelial cells after lens surgery can inhibit the formation of secondary cataract. In addition, maintaining the correct proportion of extracellular matrix around the lens is essential for the integrity and the ability of the lens to regulate outgoing signals.

### Collagen

Some of the main components of the extracellular matrix of the lens are different types of collagens (Additional file 9: Table S1). These proteins are part of the capsular bag and are essential to maintain the integrity of the lens. Interestingly, there is only one type of collagen differentially expressed between the lens and non-lens tissues (Additional file 9: Table S1, column 4). Collagen proteins are down-regulated during Sparc-related cataract, which might reflect an interaction between the Sparc glycoprotein (Additional file 9: Table S1, column 6). Sparc protein is very important when it comes to extracellular matrix. It can interact with collagen and other proteins of the extracellular matrix. It can also have an effect in the production of collagen. Thus, the Sparc-null lens make less collagen, which in turn can result in weaker lens capsule and disruption of its normal osmosis. Some collagen proteins are up-regulated during regeneration of the lens (Additional file 9: Table S1, column 9). Also, mutations in collagen genes have been shown to link with diseases, as discussed in the introduction.

### Ribosomal proteins - protein synthesis

All the cells require ribosomal proteins and the protein synthesis machinery in order to create proteins that are needed for cell survival (Additional file 10: Table S3). It has been shown that there is differential regulation of these ‘housekeeping’ proteins, and they may have other roles in the cell for different conditions [255-257]. As shown in Additional file 10: Table S3, column 8, there are a lot of ribosomal proteins that are down-regulated during age-related cataract, which might reflect the disorganization of crucial networks leading to lens opacification. On the other hand, there are mitochondrial ribosomal proteins that are down-regulated during regeneration, which can reflect stress in the lens (Additional file 10: Table S3, column 10). Inactivation of ribosomal proteins S3, S8, S11 and of translation initiation factors is linked to the increase of life span in *Caenorhabditis elegans*, which means a possible energy conservation by controlling the rate of protein synthesis [258]. Such a mechanism might be more wide-spread in diseases as well.

## Competing interests

The authors declare that they have no competing interests.

## Authors' contributions

KS and PAT conceived the idea, analyzed the data and co-wrote the paper. Both authors read and approved the final manuscript.

## Supplementary Material

Additional file 1 **Table S2. **List of all genes and their expression in different conditions of the lens. Click here for file

Additional file 2 **Table S4. **List of genes related to crystallins and heat shock proteins and their expression in different conditions of the lens. Click here for file

Additional file 3 **Table S3. **List of genes that are present in two or more different conditions of the lens. Click here for file

Additional file 4 **Table S6. **List of genes related to cytochromes and their expression in different conditions of the lens. Click here for file

Additional file 5 **Table S7. **List of genes related to transcriptional factors and their expression in different conditions of the lens. Click here for file

Additional file 6 **Table S8. **List of genes related to immunity and their expression in different conditions of the lens. Click here for file

Additional file 7 **Table S9. **List of genes related to growth factors and their expression in different conditions of the lens. Click here for file

Additional file 8 **Table S10. **List of genes related to metalloproteinases and cathepsins and their expression in different conditions of the lens. Click here for file

Additional file 9 **Table S1. **List of genes related to collagen and their expression in different conditions of the lens. Click here for file

Additional file 10 **Table S3. **List of genes related to ribosomal proteins and translation and their expression in different conditions of the lens. Click here for file
